# Decreased B lymphocytes subpopulations are associated with higher atherosclerotic risk in elderly patients with moderate-to-severe chronic kidney diseases

**DOI:** 10.1186/s12882-021-02613-6

**Published:** 2021-11-30

**Authors:** Jieshan Lin, Bin Tang, Zhanwu Feng, Wenke Hao, Wenxue Hu

**Affiliations:** 1 Department of Nephrology, Guangdong Provincial People’s Hospital, Guangdong Academy of Medical Sciences, Guangdong Provincial Geriatrics Institute, Guangzhou, 510080 China; 2 Department of Nephrology, Blood Purification Center, Zhongshan City People’s Hospital, Zhongshan, 528403 China; 3 Department of Ultrasound, Guangdong Provincial People’s Hospital, Guangdong Academy of Medical Sciences, Guangzhou, 510080 China

**Keywords:** B cells, Atherosclerosis, Intima-media thickness, Elderly, Chronic kidney disease

## Abstract

**Aim:**

Cardiovascular diseases (CVD) are the leading cause of death in patients with chronic kidney disease (CKD), and the risk of CVD increases with reductions in renal function. This study aims to investigate the potential roles of B lymphocyte populations in subclinical atherosclerosis (measured by intima-media thickness, IMT) and prognosis in elderly patients with moderate-to-severe CKD.

**Methods:**

In this study, a total of 219 patients (143 moderate-to-severe CKD patients with stage 3–4 and 76 non-CKD controls) were recruited. B cell subsets: CD19(+)CD5(+) and CD19(+)CD5(−) B cells were analyzed by flow cytometry. Intima-media thickness (IMT) was measured by ultrasound. Correlations between the B cell subsets with IMT and clinical outcome was analyzed.

**Results:**

CKD patients showed increased IMT (*P* = 0.006). The level of CD19(+)CD5(+) and CD19(+)CD5(−) B cells were decreased in CKD patients. Correlation analysis showed that IMT was positively correlated with systolic blood pressure, protein/creatinine ratio and diabetes (*P* < 0.05), and were negatively correlated with CD19(+)CD5(+) and CD19(+)CD5(−) B lymphocytes (*P* < 0.05). Stepwise multiple regression analysis showed that CD19(+)CD5(−) B cells had a significant independent association with IMT (*P* < 0.05). IMT was increased in lower level of total CD19(+) B cells (≤ 0.06 × 10^9^ /L) and CD19(+)CD5(−) B cells (≤ 0.05 × 10^9^ /L) (*P* < 0.05). Kaplan-Meier analysis showed that patients with lower levels of CD19(+)CD5(+) and CD19(+)CD5(−) B cells exhibited worse survival (*P* < 0.05). Cox regression analysis showed that patients with lower CD19(+)CD5(+) and CD19(+)CD5(−) B cells counts have a higher risk of all-cause mortality (*P* < 0.05).

**Conclusions:**

Our results showed that decreased CD19(+)CD5(+) and CD19(+)CD5(−) B lymphocytes were correlated with atherosclerosis and worse survival, which indicates that B lymphocytes might involve in atherosclerosis and associated the prognosis of elderly patients with moderate-to-severe CKD.

## Introduction

Cardiovascular diseases (CVD) are the leading cause of death in patients with chronic kidney disease (CKD), and the risk of CVD increases with reductions in renal function [1]. It is well known that CKD on hemodialysis is closely associated with accelerated atherosclerosis. However, recent studies have shown that increased atherosclerosis risk was actually observed in the early stages of CKD and remains stable thereafter [2]. Atherosclerosis is intimately interconnected with the immune system and recently, the influence of immune abnormalities on the pathogenesis of atherosclerosis and the possible discovery of new treatment methods have been increasingly studied [3]. Several studies have indicated that T cells promote atherosclerosis, whereas B cells may have a protective role [4, 5].

B cells can broadly be divided into 2 subsets: CD19(+)CD5(+) B cells and CD19(+)CD5(−) B cells [5]. CD19(+)CD5(+) B cells are largely fetal liver derived and considered to be elements of the innate immune system. They patrol mucosal surfaces, provide immediate defense and antigen capture, as well as modulate and systematically transport invading bacteria. CD19(+)CD5(+) B cells may have a protective effect on atherosclerosis that is largely mediated by the production of IgM antibodies. And these antibodies constitute a readily available pool of immunoglobulins for use against a variety of infections before specific high-affinity antibodies are produced [6]. CD19(+)CD5(−) B cells are bone marrow derived and differentiate into plasma cells and produce high affinity, class-switched antibodies in a T cell dependent manner that are responsible for developing an adaptive response [7].

Studies have suggested B cells may play the protective effect on atherosclerosis. Clinical studies have also reported a negative association between (IgM-producing) memory B cells and risk of CVD [8, 9]. In addition, distinct knockout mouse models targeting B cell subsets differentially have revealed the antiatherogenic potentials of CD19(+)CD5(+) and CD19(+)CD5(−) B cells [10–12]. Our previous data and some other results showed that CD19(+)CD5(+) and CD19(+)CD5(−) B cells exhibited a significantly negative correlation with the progression of CKD in elderly patients and patients with decreased B cell counts had a higher risk of CVD mortality [13, 14]. However, the roles of B cell subpopulations in atherosclerosis of elderly patients with CKD are unclear.

Ultrasonic measurement of the thickness of the carotid intima-media thickness (IMT) is a noninvasive and reliable tool to assess asymptomatic atherosclerosis and increased IMT is an independent predictor of future cardiovascular risk [15]. IMT is increased in CKD patients and may help to predict patients at higher risk of future CVD [16]. Therefore, the aim of this study was to investigate the potential roles of peripheral blood B lymphocyte populations in subclinical atherosclerosis (measured by IMT) and prognosis in elderly patients with moderate-to-severe CKD.

## Materials and methods

### Patients

A total of 143 patients with moderate-to-severe CKD (stage 3–4) aged ≥65 years were retrospectively studied in Guangdong Provincial People’s Hospital from January 2010 to December 2018. CKD is defined as abnormalities of kidney structure or function, present for > 3 months. The criteria for CKD are as follows: (1) decreased GFR (for > 3 months): GFR < 60 ml/min/1.73 m^2^, and/ or (2) markers of kidney damage (> 1 for > 3 months): albuminuria, electrolyte, urinary sediment abnormalities, and other abnormalities due to abnormalities detected by histology, tubular disorders, structural abnormalities detected by imaging, history of kidney transplantation. The estimated glomerular filtration rate (eGFR) was calculated using CKD-EPI equation, and the definition of stage 3 was eGFR from 30 to 59 ml/min/1.73 m^2^, stage 4 was eGFR from 15 to 29 ml/min/1.73m^2^. Patients with acute kidney injury, malignancy, active infection, thyroid malfunction, heart failure or on immunosuppressive drugs were excluded.

The control group (76 patients) without CKD included patients hospitalized for hypertension, prostatic hyperplasia and osteoporosis. For all patients, we recorded clinical data, including age and sex, serum creatinine (SCr), protein/creatinine ratio, serum albumin (ALB), cholesterol, triglyceride, white blood cell (WBC), lymphocytes and neutrophil levels; and comorbidities such as diabetes and hypertension. SCr was measured by the enzymatic method. The study involving human participants was approved by the Ethical Committee of Guangdong Provincial People’s Hospital.

### Clinical outcome

Outcome of this study was all-cause mortality. All patients were followed until the date of their last visit, and the median follow-up duration was 41.5 months.

### Estimation of intima–media thickness (IMT)

The IMT was measured bilaterally at the far wall over the distal 1.5-cm segment of the common carotid artery by using a high-resolution 4- to 13-MHz linear-array transducer system. For each subject, the mean IMT was computed as the average IMT on both sides.

### Flow Cytometry analysis (FCM)

**Fig. 1:**
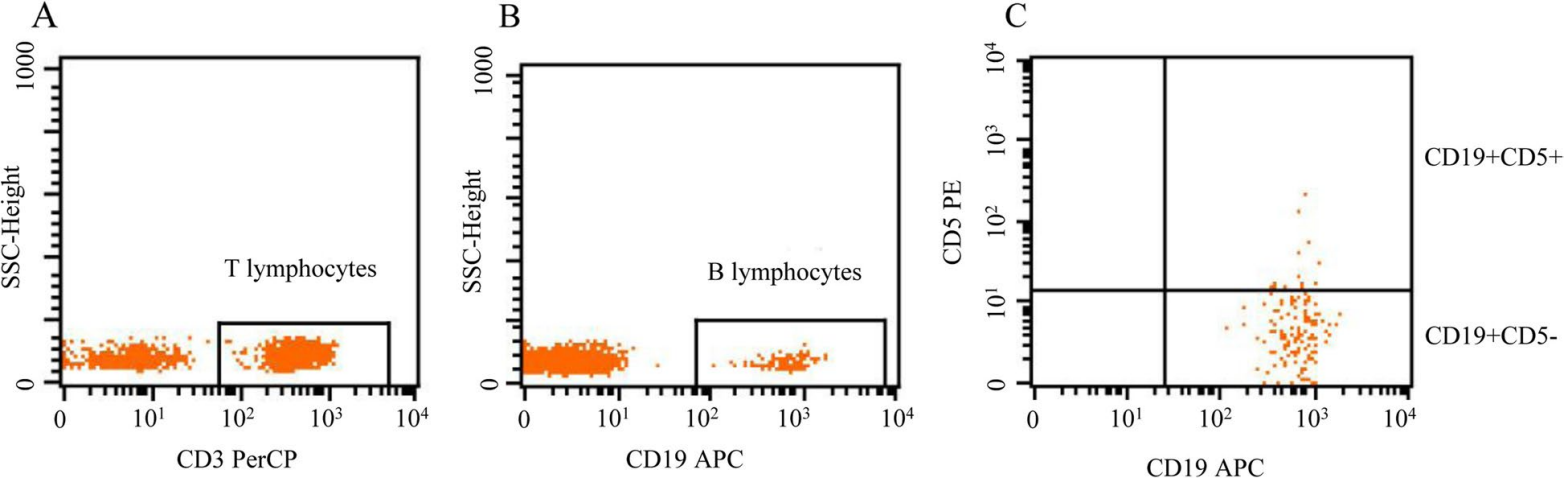
Flow cytometric analysis of lymphocytes, including T lymphocytes (CD3+) and B lymphocytes (CD19+). B cells were divided into CD19(+)CD5(+) B cells and CD19(+)CD5(−) B cells

Peripheral blood lymphocyte populations (CD3(+) T lymphocytes, total CD19(+) B lymphocytes, CD19(+)CD5(+) B lymphocytes, and CD19(+)CD5(−) B lymphocytes) were analyzed by flow cytometry. Blood samples obtained by venipuncture were collected in EDTA anticoagulant. 10 μl of anti-human CD3-PerCP, CD19-APC, and CD5-PE antibodies were added to 100 μl of whole blood and incubated for 20 min at 4 °C in the dark. A total of 2 ml of red blood cell lysis buffer was added before staining to each tube, vortexed well, and incubated for 10 min at room temperature in darkness. Then, the cells were washed twice with phosphate buffer saline (PBS), and then discarded the supernatant. Last, 20,000 cells were acquired by using FACS (BD USA) and were analyzed using Cellquest-Pro analysis software to determine the subpopulation counts (Fig. 1).

### Statistical analysis

Data are expressed as mean ± SEM. Statistical analyses were performed using SPSS version 20.0 (Chicago, IL, USA). Continuous variables were analyzed by the Wilcoxon rank-sum test. The correlations between IMT and clinical data were performed using the Spearman’s test. Stepwise multiple regression analysis was performed to evaluate the independent predictor of IMT. The X-tile software version 3.6.1 (Yale University, New Haven, USA) was used to determine the optimal cutoff points of IMT and different B cells based on the outcome [17]. And the outcome of this study was all-cause mortality. The optimal cutoff points of IMT, total CD19(+), CD19(+)CD5(+) and CD19(+)CD5(−) B cells were 1.0 cm, 0.06 × 10^9^ /L, 0.03 × 10^9^ /L and 0.05 × 10^9^/L, respectively.

The Kaplan–Meier survival analysis method with the log-rank test was used to compare survival times and Cox regression analysis was used for univariate and multivariate analyses. All *p* values were calculated two-sided and a value of < 0.05 was considered significant.

## Results

### Patient and demographic details

The clinical and demographic data of the study subjects are shown in Table 1. There were 219 patients entered in the final analysis: 76 control subjects and 143 patients with CKD stage 3–4. The majority of patients were males. 86.01% of the CKD patients had hypertension and 42.66% were with diabetic.

**Table 1:** Clinical characteristics and laboratory parameters of older patients with or without CKD

	Control subjects	CKD patients	*P* value
Number of cases	76	143	
Age, years	80.32 ± 4.34	82.70 ± 4.89	< 0.001^*^
Gender (M/F)	60/16	115/28	< 0.001^*^
Hypertension	60.53%	86.01%	< 0.001^*^
Systolic blood pressure, mmHg	134.37 ± 16.78	143.66 ± 16.62	< 0.001^*^
Diastolic blood pressure, mmHg	71.17 ± 9.09	74.66 ± 10.22	0.005^*^
Diabetes	23.68%	42.66%	0.005^*^
SCr (μmol/L)	77.64 ± 11.47	145.55 ± 50.42	< 0.001^*^
eGFR CKD − EPI (ml/min/1.73 m^2^)	78.32 ± 10.65	40.98 ± 13.49	< 0.001^*^
Albumin (g/L)	35.00 ± 33.72	33.72 ± 4.97	0.075
protein/creatinine ratio (mg/g Cr)	115.17 ± 98.40	776.87 ± 1587.69	< 0.001^*^
Cholesterol (mmol/L)	4.19 ± 0.90	4.43 ± 1.29	0.401
Triglyceride (mmol/L)	1.12 ± 0.60	1.36 ± 0.89	0.040^*^
IMT (cm)	0.94 ± 0.16	1.01 ± 0.17	0.006^*^
WBC (10^9^ /L)	6.17 ± 1.95	6.43 ± 1.92	0.247
Neutrophil (10^9^ /L)	3.75 ± 1.51	4.20 ± 1.59	0.027^*^
T lymphocytes (10^9^ /L)	1.09 ± 0.44	0.33 ± 0.21	0.175
Total CD19(+) B lymphocytes (10^9^ /L)	0.22 ± 0.36	0.15 ± 0.11	0.059
CD19(+)CD5(+) B lymphocytes (10^9^ /L)	0.10 ± 0.33	0.05 ± 0.07	0.040^*^
CD19(+)CD5(−) B lymphocytes (10^9^ /L)	0.12 ± 0.09	0.10 ± 0.06	0.088
Number of deaths	9	46	0.001^*^
Follow up time	50.37 ± 31.59	38.46 ± 29.56	0.009^*^

*eGFR* Estimated glomerular filtration rate, *SCr* Serum creatinine, *IMT* Intima–media thickness, *WBC* White blood cell

### Intima–media thickness and B cell subpopulations

The common carotid artery IMT was 1.01 ± 0.17 cm in CKD patients and 0.94 ± 0.16 cm in the control group (*P* = 0.006). Comparison of B cells in the two groups revealed remarkable differences, and the number of B lymphocyte subpopulation was decreased in CKD patients. The levels of total CD19(+), CD19(+)CD5(+) and CD19(+)CD5(−) B lymphocytes in CKD patients were significantly lower than those in the control group (Table 1).

### The correlation between IMT and clinical data

Spearman’s analysis showed that IMT was positively correlated with systolic blood pressure (R = 0.282, *P* = 0.001), protein/creatinine ratio (*R* = 0.253, *P* = 0.016) and diabetes (R = 0.267, *P* = 0.001). We further analyzed the association of IMT and B cells. Our data revealed that IMT was negatively correlated with total CD19(+) B lymphocytes (*R* = -0.209, *P* = 0.012), CD19(+)CD5(+) B lymphocytes (*R* = -0.195, *P* = 0.020) and CD19(+)CD5(−) B lymphocytes (*R* = -0.208, *P* = 0.013). In contrast, in the control group, only diabetes (*R* = 0.243, *P* = 0.034) exhibited positive correlation with IMT (Table 2). In stepwise multiple regression analysis, CD19(+)CD5(−) B cells was found to have a significant independent association with IMT (Standardized beta:-0.226, *P* = 0.033) (Table 3).

**Table 2:** Correlation between IMT with the clinical data of older patients with or without CKD

Variable	IMT (Control)	IMT (CKD)
Age	*R* = -0.071	*R* = 0.147
	*P* = 0.545	*P* = 0.080
Systolic blood pressure	*R* = 0.063	*R* = 0.282
	*P* = 0.589	*P* = 0.001^*^
Diastolic blood pressure	*R* = -0.102	*R* = -0.022
	*P* = 0.381	*P* = 0.792
Diabetes	*R* = 0.243	*R* = 0.267
	*P* = 0.034^*^	*P* = 0.001^*^
SCr	*R* = 0.013	*R* = 0.036
	*P* = 0.914	*P* = 0.671
Protein/creatinine ratio	*R* = 0.087	*R* = 0.253
	*P* = 0.593	*P* = 0.016^*^
Cholesterol	*R* = -0.067	*R* = -0.062
	*P* = 0.565	*P* = 0.460
Triglyceride	*R* = -0.032	*R* = -0.020
	*P* = 0.783	*P* = 0.815
WBC	*R* = 0.182	R = 0.068
	*P* = 0.115	*P* = 0.421
Neutrophil	*R* = 0.204	*R* = 0.054
	*P* = 0.077	*P* = 0.524
Total CD19(+) B lymphocytes	R = -0.087	R = -0.209
	*P* = 0.453	*P* = 0.012^*^
CD19(+)CD5(+) B lymphocytes	*R* = -0.039	*R* = -0.195
	*P* = 0.740	*P* = 0.020^*^
CD19(+)CD5(−) B lymphocytes	*R* = -0.057	*R* = -0.208
	*P* = 0.628	*P* = 0.013^*^

*IMT* Intima–media thickness, *CKD* Chronic kidney disease, *SCr* Serum creatinine, *WBC* White blood cell

**Table 3:** Stepwise multiple regression analysis showing the independent association of CD19(+)CD5(−) B cells with IMT

	Unstandardized B (SE)	Standardized beta	*P* value	95% confidence interval for B	Tolerance
CD19(+)CD5(−) B cells (10^9^ /L)	−0.588 (0.271)	−0.226	0.033	−1.126, −0.050	1.000

Systolic blood pressure, diabetes, protein/creatinine ratio, CD19(+) B cells and CD19(+)CD5(+) B cells were also included, but they were not significantly associated with IMT. IMT, intima–media thickness

### Comparison of IMT between low and high level of different B cells in the total cohort and CKD group

**Fig. 2:**
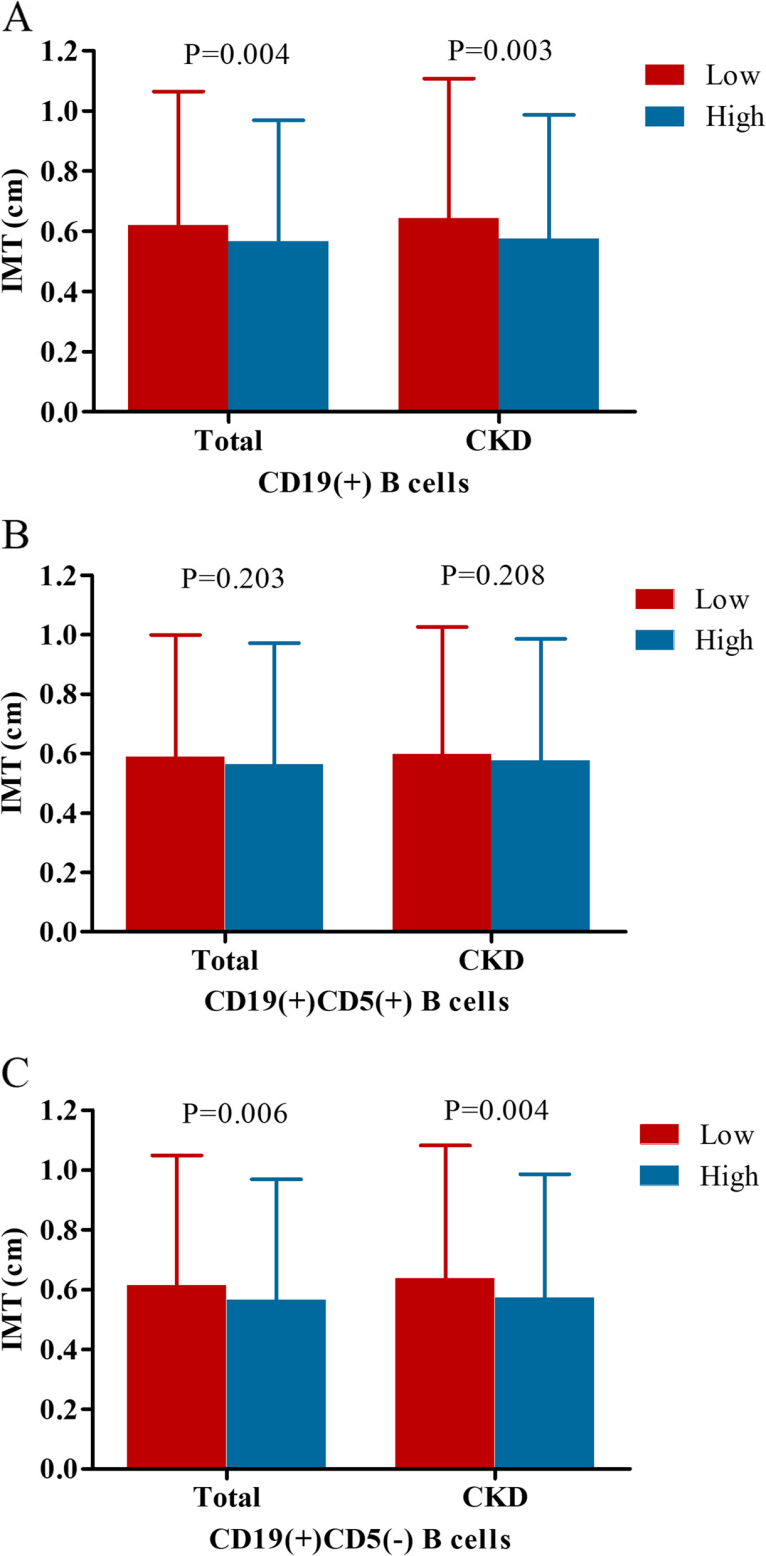
Comparison of intima-media thickness (IMT) between low and high level of different B cells in the total cohort and CKD group. **A** Total CD19(+) B cells: low: ≤0.06 × 10^9^ /L and high: > 0.06 × 10^9^ /L; **B** CD19(+)CD5(+) B cells: low: ≤0.03 × 10^9^ /L and high: > 0.03 × 10^9^ /L; (C) CD19(+)CD5(−) B cells: low: ≤0.05 × 10^9^ /L and high: > 0.05 × 10^9^ /L.

We used X-tile software to determine the optimal cutoff points of total CD19(+), CD19(+)CD5(+) and CD19(+)CD5(−) B cells based on the outcome. And the cutoff points for the grouping of total CD19(+), CD19(+)CD5(+), and CD19(+)CD5(−) B cells were 0.06 × 10^9^ /L, 0.03 × 10^9^ /L and 0.05 × 10^9^ /L, respectively. As shown in Fig. 2, IMT was increased in the lower level of total CD19(+) and CD19(+)CD5(−) B cells. IMT was increased in total CD19(+) B cells ≤0.06 × 10^9^ /L in the total cohort (1.06 ± 0.18 vs. 0.97 ± 0.16, *P* = 0.004) and CKD group (1.11 ± 0.18 vs. 0.99 ± 0.16, *P* = 0.003). Similarly, IMT was also increased in CD19(+)CD5(−) B cells ≤0.05 × 10^9^ /L in the total cohort (1.05 ± 0.18 vs. 0.97 ± 0.16, *P* = 0.006) and CKD group (1.08 ± 0.19 vs. 0.99 ± 0.16, P = 0.004). Though IMT was increased in CD19(+)CD5(+) B cells ≤0.03 × 10^9^ /L, the difference was not significant (*P* > 0.05).

### Association of IMT and different B cells with overall survival

**Fig. 3:**
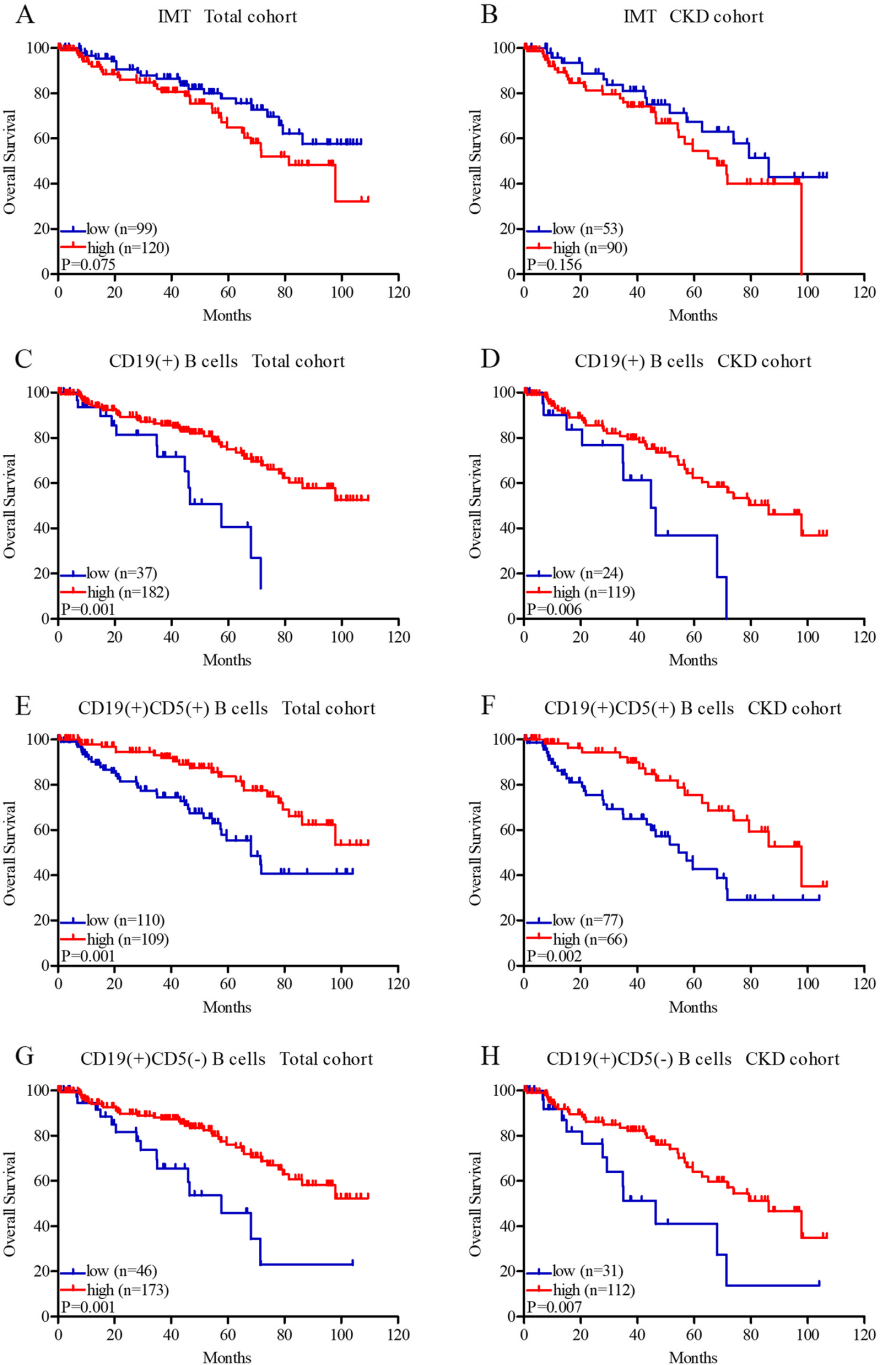
Kaplan–Meier survival curves of intima-media thickness (IMT), total CD19(+) B cells, CD19(+)CD5(+) B cells, and CD19(+)CD5(−) B cells in the total cohort (**A**, **C**, **E**, **G**) and CKD group (**B**, **D**, **F**, **H**). IMT, total CD19(+) B cells, CD19(+)CD5(+) B cells, and CD19(+)CD5(−) B cells were divided into two groups (low and high) according to the optimal cutoff points, IMT: low: ≤1.0 cm and high: > 1.0 cm; total CD19(+) B cells: low: ≤0.06 × 10^9^ /L and high: > 0.06 × 10^9^ /L; CD19(+)CD5(+) B cells: low: ≤0.03 × 10^9^ /L and high: > 0.03 × 10^9^ /L; CD19(+)CD5(−) B cells: low: ≤0.05 × 10^9^ /L and high: > 0.05 × 10^9^ /L.

As mentioned above, patients were divided into two groups according to the optimal cutoff points, and the cutoff point of IMT was 1.0 cm. Kaplan-Meier analysis showed that patients with IMT ≤1.0 cm present longer survival in the total cohort (83.92 ± 4.03 vs. 73.99 ± 4.48 months, *P* = 0.075, Fig. 3A) and CKD group (75.64 ± 5.83 vs. 63.35 ± 4.60, *P* = 0.156 months, Fig. 3B), but the difference was not statistically significant. Patients with total CD19(+) B cells > 0.06 × 10^9^ /L showed prolonged survival in the total cohort (50.07 ± 4.53 vs. 83.51 ± 3.23 months, *P* = 0.001, Fig. 3C) and CKD group (45.35 ± 6.27 vs. 73.29 ± 4.12, *P* = 0.006 months, Fig. 3D). The group with CD19(+)CD5(+) B cells > 0.03 × 10^9^ /L also had better survival in the total cohort (67.27 ± 4.55 vs. 89.11 ± 3.65 months, *P* = 0.001, Fig. 3E) and CKD group (57.83 ± 5.35 vs. 81.43 ± 4.80 months, *P* = 0.002, Fig. 3F). The results of CD19(+)CD5(−) B cells were similar, higher levels of CD19(+)CD5(−) B cells (> 0.05 × 10^9^ /L) exhibited better survival in the total cohort (57.57 ± 7.16 vs. 83.86 ± 3.26 months, *P* = 0.001, Fig. 3G) and CKD group (49.18 ± 7.98 vs. 73.73 ± 4.16 months, *P* = 0.007, Fig. 3H).

### Analysis of prognostic factors

In the total cohort, univariate analysis showed that age, hypertension, systolic pressure, diabetes, SCr, CD19(+)CD5(+) B cells and CD19(+)CD5(−) B cells were prognostic factors for overall survival in the total cohort, multivariate Cox regression analysis showed that age (HR = 1.100, 95%CI: 1.030–1.174, *P* = 0.004), systolic pressure (HR = 1.026, 95%CI: 1.007–1.046, *P* = 0.008), SCr (HR = 1.009, 95%CI: 1.005–1.014, *P* < 0.001) and CD19(+)CD5(−) B cells≤0.05 × 10^9^ /L (HR = 2.821, 95%CI: 1.482–5.370, *P* = 0.002) were factors significantly associated with a higher risk of all-cause mortality (Table 4).

**Table 4:** Univariate and multivariate analyses for all-cause mortality in the total cohort

Variables	Univariate analysis		Multivariate analysis	
	HR (95% CI)	*P* value	HR (95% CI)	*P* value
Age (year)	1.128 (1.056–1.205)	**< 0.001**	1.100 (1.030–1.174)	**0.004**
Female Sex	0.951 (0.447–2.026)	0.897		
Hypertension	2.557 (1.091–5.991)	**0.031**		
Systolic pressure (mmHg)	1.031 (1.014–1.048)	**< 0.001**	1.026 (1.007–1.046)	**0.008**
Diastolic pressure (mmHg)	1.023 (0.993–1.053)	0.135		
Diabetes	1.898 (1.108–3.252)	**0.020**		
SCr (μmol/L)	1.008 (1.005–1.012)	**< 0.001**	1.009 (1.005–1.014)	**< 0.001**
Protein/creatinine ratio (mg/g Cr)	1	0.161		
Triglyceride (mmol/L)	1.132 (0.864–1.482)	0.368		
IMT (cm)	1.881 (0.375–9.433)	0.442		
Neutrophil (10^9^ /L)	1.056 (0.918–1.215)	0.443		
CD19(+)CD5(+) B lymphocytes (10^9^/L)				
≤ 0.03	2.472 (1.425–4.289)	**0.001**		
> 0.03				
CD19(+)CD5(−) B lymphocytes (10^9^/L)				
≤ 0.05	2.665 (1.451–4.895)	**0.002**	2.821 (1.482–5.370)	**0.002**
> 0.05				

*Notes*: *IMT* Intima–media thickness, *SCr* Serum creatinine

In the CKD patients, univariate analysis showed that age, systolic pressure, SCr, CD19(+)CD5(+) B cells and CD19(+)CD5(−) B cells were prognostic factors for overall survival, multivariate Cox regression analysis showed that age (HR = 1.088, 95%CI: 1.015–1.166, *P* = 0.017), SCr (HR = 1.006, 95%CI: 1.001–1.011, *P* = 0.018) and CD19(+)CD5(+) B cells≤0.03 × 10^9^ /L (HR = 2.303, 95%CI: 1.241–4.273, *P* = 0.008) were factors significantly associated with a higher risk of all-cause mortality (Table 5).

**Table 5:** Univariate and multivariate analyses for all-cause mortality in the CKD patients

Variables	Univariate analysis		Multivariate analysis	
	HR (95% CI)	*P* value	HR (95% CI)	*P* value
Age (year)	1.080 (1.012–1.153)	**0.020**	1.088 (1.015–1.166)	**0.017**
Female Sex	0.886 (0.373–2.107)	0.784		
Hypertension	1.508 (0.537–4.240)	0.436		
Systolic pressure (mmHg)	1.020 (1.001–1.040)	**0.044**		
Diastolic pressure (mmHg)	1.005 (0.974–1.036)	0.764		
Diabetes	1.689 (0.928–3.074)	0.086		
SCr (μmol/L)	1.005 (1.000–1.010)	**0.035**	1.006 (1.001–1.011)	**0.018**
Protein/creatinine ratio (mg/g Cr)	1	0.556		
Triglyceride (mmol/L)	1.000 (0.751–1.332)	0.998		
IMT (cm)	1.462 (0.284–7.516)	0.649		
Neutrophil (10^9^ /L)	0.985 (0.833–1.164)	0.985		
CD19(+)CD5(+) B lymphocytes (10^9^ /L)				
≤0.03	2.542 (1.376–4.699)	**0.003**	2.303 (1.241–4.273)	**0.008**
> 0.03				
CD19(+)CD5(−) B lymphocytes (10^9^ /L)				
≤0.05	2.457 (1.257–4.805)	**0.009**		
> 0.05				

*Notes*: *CKD* Chronic kidney disease, *IMT* Intima–media thickness, *SCr* Serum creatinine

## Discussion

In CKD subjects, cardiovascular disease is a major cause of morbidity and mortality, account for 40% of deaths among patients with end-stage renal disease (ESRD) [18]. CKD accelerates atherosclerosis via augmentation of inflammation, perturbation of lipid metabolism, and other mechanisms [4]. It has been well established that the innate and adaptive immunity contribute to the development of atherosclerosis, and B cells have emerged as important modulators of inflammatory effects in atherosclerosis [5]. B lymphocytes are generated from hematopoietic stem cells in bone marrow. Murine B cells are broadly divided into CD19(+)CD5(+) and CD19(+)CD5(−) subsets, CD19(+)CD5(+) B cells secrete IgM and IgA while CD19(+)CD5(−) B cells produce IgG [6]. Previous studies have shown that B lymphocytes have a protective effect on atherosclerosis [10–12]. However, few studies have analyzed the relationship between B cell subsets and atherosclerosis of moderate-to-severe CKD patients in the elderly.

In this study, we found that IMT was positively correlated with systolic blood pressure, protein/creatinine ratio and diabetes. Hypertension is a major risk factor for the development of atherosclerosis. In hypertension, changes in microcirculation can cause endothelial dysfunction, then promotes the formation of atherosclerotic plaque [19]. Atherosclerosis is the most important cause of morbidity and mortality in CKD. And Sumida et al. [20] showed that carotid artery calcification was significantly associated with proteinuria in ESRD patients. Diabetic macroangiopathy, atherosclerosis secondary to diabetes, is characterized by the alterations in vascular homeostasis due to endothelial and vascular smooth muscle cell dysfunction [21].

In addition, our results showed that CD19(+)CD5(+) and CD19(+)CD5(−) B lymphocytes were negatively correlated IMT. B cells are divided into CD19(+)CD5(+) and CD19(+)CD5(−) subsets. CD19(+)CD5(+) B cells have been reported to protect from atherosclerosis by the production of IgM antibodies. IgM antibodies have the capacity to recognize apoptotic cells and Oxidized LDL (OxLDL) and limit foam cell formation and OxLDL-induced endothelial activation [5]. Gruber et al. [12] studied the Siglec-G-deficient mice and found that CD5(+) B cells derived natural IgM had an effect on decreasing levels of OxLDL and oxidation-specific epitopes, so could reduce atherosclerosis. In addition, previous studies have also showed that CD19(+)CD5(+) B cells were significant source of interleukin 10 (IL10), which also exert atheroprotection [22]. Our results showed that the levels of CD19(+)CD5(+) B cells was significantly decreased in moderate-to-severe CKD patients and IMT was increased in the lower levels of CD19(+)CD5(+) B cells, that could explain why patients with CKD exhibit accelerated development of atherosclerosis.

Moreover, our data also showed that CD19(+)CD5(−) B lymphocytes were negatively correlated IMT and IMT was significantly increased in the lower levels of CD19(+)CD5(−) B cells. Moreover, stepwise multiple regression analysis showed that CD19(+)CD5(−) B cells had a significant independent association with IMT. Genomic data showed that the survival of CD19(+)CD5(−) B cells was mainly dependent on the B cell-activating factor (BAFF) receptor pathway [23]. Tsiantoulas et al. [24] studied Apoe−/− and Ldlr−/− mice and found that anti-BAFF antibody treatment could deplete CD19(+)CD5(−) B cells and increased atherosclerosis. In CKD patients, the uremic environment may promote resistance to BAAF-mediated signals and interfere the maturation of transitional B cells to mature B cells [25]. Therefore, decreased CD19(+)CD5(−) B cells might be associated with the uremic environment and was correlated with atherosclerosis.

Furthermore, our study showed that IMT was increased in lower levels of CD19(+)CD5(+) and CD19(+)CD5(−) B cells, and moderate-to-severe CKD patients with lower levels of CD19(+)CD5(+) and CD19(+)CD5(−) B cells exhibited worse survival. Cox regression analysis showed that patients with lower CD19(+)CD5(+) and CD19(+)CD5(−) B cells counts have a higher risk of all-cause mortality. Progressive loss of renal function is associated with immune deficiency, and account for the large proportion of morbidity and mortality [26]. Molina et al. [13] showed that patients with lower levels of B cells had a higher risk of CVD and all-cause mortality. These data indicated that higher levels of B cells may play a protective role in CKD and had a potential effect of inhibiting atherosclerosis.

However, our study also had certain limitations. Firstly, this was a retrospective study. Secondly, we did not explore the mechanisms of different B lymphocytes in atherosclerosis. Further studies should be carried out to explore these results.

In conclusion, our results showed that decreased CD19(+)CD5(+) and CD19(+)CD5(−) B lymphocytes were correlated with atherosclerosis and worse clinical outcome, which indicated that B lymphocytes may be involved in pathogenesis of atherosclerosis and associated with prognosis in moderate-to-severe CKD.

## Data Availability

The datasets are available from the corresponding author on reasonable request.

## Abbreviations

CVD: Cardiovascular diseases
CKD: Chronic kidney disease
IMT: Intima-media thickness
eGFR: Estimated glomerular filtration rate
SCr: Serum creatinine
ALB: Serum albumin
WBC: White blood cell
PBS: Phosphate buffer saline
ESRD: End-stage renal disease
OxLDL: Oxidized LDL
IL10: Interleukin 10
BAFF: B cell-activating factor
